# Physical evaluation of an ultra-high-resolution CT scanner

**DOI:** 10.1007/s00330-019-06635-5

**Published:** 2020-02-10

**Authors:** Luuk J. Oostveen, Kirsten L. Boedeker, Monique Brink, Mathias Prokop, Frank de Lange, Ioannis Sechopoulos

**Affiliations:** 1 Department of Radiology and Nuclear Medicine, Radboud University Medical Center, P.O. Box 9101 (Route 766), 6500 HB Nijmegen, The Netherlands; 2 Canon Medical Systems Corporation, Otawara, Japan

**Keywords:** Physics, Phantoms, Imaging, Multi-detector computed tomography

## Abstract

**Objectives:**

To evaluate the technical performance of an ultra-high-resolution CT (UHRCT) system.

**Methods:**

The physico-technical capabilities of a novel commercial UHRCT system were assessed and compared with those of a current-generation multi-detector (MDCT) system. The super-high-resolution (SHR) mode of the system uses 0.25 mm (at isocentre) detector elements (dels) in the in-plane and longitudinal directions, while the high-resolution (HR) mode bins two dels in the longitudinal direction. The normal-resolution (NR) mode bins dels 2 × 2, resulting in a del-size equivalent to that of the MDCT system. In general, standard procedures and phantoms were used to perform these assessments.

**Results:**

The UHRCT MTF (10% MTF 4.1 lp/mm) is twice as high as that of the MDCT (10% MTF 1.9 lp/mm), which is comparable to the MTF in the NR mode (10% MTF 1.7 lp/mm). The width of the slice sensitivity profile in the SHR mode (FWHM 0.45 mm) is about 60% of that of the MDCT (FWHM 0.77 mm). Uniformity and CT numbers are within the expected range. Noise in the high-resolution modes has a higher magnitude and higher frequency components compared with MDCT. Low-contrast visibility is lower for the NR, HR and SHR modes compared with MDCT, but about a 14%, for NR, and 23%, for HR and SHR, dose increase gives the same results.

**Conclusions:**

HR and SHR mode scanning results in double the spatial resolution, with about a 23% increase in dose required to achieve the same low-contrast detectability.

**Key Points:**

*• Resolution on UHRCT is up to twice as high as for the tested MDCT.*

*• With abdominal settings, UHRCT needs higher dose for the same low-contrast detectability as MDCT, but dose is still below achievable levels as defined by current diagnostic reference levels.*

*• The UHRCT system used in normal-resolution mode yields comparable resolution and noise characteristics as the MDCT system.*

**Electronic supplementary material:**

The online version of this article (10.1007/s00330-019-06635-5) contains supplementary material, which is available to authorized users.

## Introduction

Advances in multi-detector computed tomography (MDCT) technology have continued over recent years. New detector hardware has resulted in the introduction of wider detectors [1], new electronic designs with lower noise [2] and, more recently, smaller detector elements [3]. The ability to visualise anatomy and pathology in greater detail has the potential to benefit the imaging of the lung, temporal bone, vasculature and stent structure, as well as visualisation of small tumours and structures [3–6]. Ultra-high spatial resolution capabilities may also lead to a reduction in artefacts such as blooming, as well as an increased ability to quantify features of anatomical and pathological structures.

While many factors, e.g. focal spot size and number of views, impact the final spatial resolution of a CT image, the maximum spatial resolution of a CT system is fundamentally dictated by the Nyquist frequency of the detector in both the midplane and longitudinal dimensions. Several approaches to reducing the detector element size in CT have been explored in the past. First, flat panel prototype volume CT scanners were constructed, but they suffered from poor low-contrast detectability and gantry rotation speed limitations preventing commercial implementation [7–10]. A second approach was the introduction of a tantalum grid over a selected number of detector elements in a conventional CT system, in order to reduce the active detector element area, yielding 250 μm resolution images. However, this approach inherently decreased the dose efficiency, limiting its utility as a general clinical system [11]. Third, a prototype fine-cell CT scanner was developed using a 312.5-μm channel thickness that achieved 27.6 lp/cm at 2% of the MTF, but with high noise [12]. Finally, photon-counting detectors permitted the measurement of individual photons and their corresponding energy, ideally eliminating the need for detector septa and grids. An ultra-high-resolution mode on a prototype photon-counting system generated by grouping detector elements into a 2 × 2 formation rather than a 4 × 4 formation for conventional resolution resulted in 250 μm × 250 μm resolution at isocentre [13]. Currently, however, the commercial development of photon-counting scanners is hampered by pulse pileup and other technical issues [14].

Recently, an ultra-high-resolution CT system (UHRCT; Aquilion Precision, Canon Medical Systems Corporation) was brought to market with a detector element size of 0.25 mm at isocentre. The purpose of this work is to perform a fundamental physics assessment of this UHRCT system, and compare it with a conventional CT system.

## Materials and methods

For this evaluation, we compared the characteristics of the UHRCT system to a current MDCT system, with comparable hardware, geometry and reconstruction modes, but without the high spatial resolution capability. The following characteristics were measured: spatial resolution, CT number accuracy, uniformity, low-contrast detectability and noise. All measurements were made using abdominal imaging protocols.

### CT scanner and acquisition

The UHRCT system used has been previously described [3]. Briefly, this system has three resolution modes: normal resolution (NR), high resolution (HR) and super-high resolution (SHR). In NR mode, the 0.25 mm detector elements, at isocentre, are read out in 2 × 2 binned mode. The detector element size is therefore 0.5 mm in-plane as well as in the longitudinal direction, comparable to current MDCT systems. In HR mode, the in-plane element size halves while in the longitudinal direction it remains the same as in NR mode. Finally, in SHR mode, the native detector element size of 0.25 mm in both directions is used. Therefore, the in-plane resolutions of the HR and the SHR modes are the same, while the resolution in the longitudinal direction for the latter is twice as high as that of the former. The UHRCT system has various focal spot sizes, the smallest being of nominal size 0.4 × 0.5 mm^2^. This focal spot size can be used in all resolution modes up to a tube current of 260 mA. The matrix size can have dimensions of 512 × 512, 1024 × 1024 or 2048 × 2048 pixels, with the latter two sizes not being available in NR mode.

For reference, measurements were performed on a current MDCT system (Aquilion One Genesis, Canon Medical Systems Corporation) with a fixed detector element size of 0.5 mm in both directions. The smallest nominal focal spot size of this system is 0.9 × 0.8 mm^2^.

The default acquisition parameters for this evaluation are given in Table 1. These parameters mimic an abdominal CT protocol as used at our institution. As stated in Table 1, all acquisitions were reconstructed using hybrid-iterative reconstruction (AIDR 3D Enhanced, FC08). The only exception is the measurement of the maximum spatial resolution, which was performed using filtered back projection and a high-resolution kernel (FC90). In all cases, the same reconstruction technique was used on both CT systems.

**Table 1:** Default parameters for the measurements performed. Deviation from these parameters for each test are noted in the corresponding test descriptions

Parameter	UHRCT	MDCT
Tube voltage (kVp)	120	120
Tube current (mA)	260	270
Effective tube current time product (mAs)	160	166
Computed tomography dose index volume (mGy)	NR 9.1 HR 11.2 SHR 11.3	9.2
Rotation time (s)	0.5	0.5
Focal spot size (mm^2^)	0.4 × 0.5	0.9 × 0.8
Scan mode, collimation (mm)	Helical, NR/HR 80 × 0.50 SHR 160 × 0.25	Helical, 80 × 0.50
Pitch	0.813	0.813
Field of view (mm)	500	500
Reconstruction method	AIDR 3D enhanced	AIDR 3D enhanced
Reconstruction kernel	FC08	FC08
Reconstruction matrix	NR 512 × 512 HR/SHR 1024 × 1024	512 × 512
Slice thickness, increment (mm)	SHR 0.25, 0.25 HR/NR 0.50, 0.50	0.5, 0.5

### Spatial resolution

The maximum spatial resolution that the UHRCT can produce was determined by measuring the modulation transfer function (MTF) using a sequential acquisition mode and filtered backprojection reconstruction. A 50-μm diameter tungsten wire fixed in a frame of balsa wood was imaged. The wire was positioned approximately 1.5 cm above the isocentre and scanned using the sequential scan mode. In this mode, only the central 4 detector slices are used and combined in one reconstructed slice. A 20-mm FOV was reconstructed using filtered backprojection and a high-resolution reconstruction kernel (FC90). The tube current was set at 100 mA. The MTF was calculated as described in Appendix A.

For the resolution measurements using hybrid-iterative reconstruction, the tungsten wire cannot be used as this reconstruction technique (AIDR 3D Enhanced) diminishes the delta pulse of such a thin wire. Therefore, the Teflon rod in the CTP401 module of the Catphan 500 phantom [15] was used. The phantom was placed such that the Teflon rod was laterally centred at five different vertical positions: at the isocentre and at 5 cm, 10 cm, 15 cm and 20 cm above the isocentre. Again, further details on the MTF calculation are provided in Appendix A.

Even though the same hybrid-iterative reconstruction algorithm and kernel are available on both systems, to exclude any possible differences that they might have internally, the MTF in the isocentre was also determined using filtered backprojection with the same kernel (FC08).

### Slice sensitivity profile

The spatial resolution in the longitudinal direction was measured using the slice sensitivity profile (SSP). For this, a 0.025-mm thick tungsten foil embedded in a polyurethane cylinder was scanned in the isocentre using the default settings as given in Table 1 and hybrid-iterative reconstruction. A 40-mm FOV was reconstructed using a slice thickness of 0.25 mm and a slice increment of 0.1 mm. Using ImageJ (version 1.52d, National Institutes of Health), the average value within a circular ROI with a 23 voxel diameter located inside the disc was plotted against the longitudinal distance. From the SSP, the full width at half maximum (FWHM) was calculated.

### CT number uniformity

Uniformity was measured using a 320-mm water phantom similar to the method proposed by the American College of Radiology (ACR) [16]. Five circular ROIs with a diameter of 32 mm were selected in the central slice, at the centre and at the 3, 6, 9 and 12 o’clock positions. The mean pixel HU values of the peripheral ROIs were compared with the central one.

### Noise

Noise appearance was measured by calculating the noise power spectrum (NPS) for different conditions using a cylindrical water phantom with a diameter of 320 mm. Default acquisition settings were used. Per setting, 80 scans with an 8-cm scan range were acquired. Three slices per scan, separated by at least 15 slices to minimise noise correlation, were used for the NPS calculation to improve statistics. The NPS was calculated using the method described in Appendix A. As a measure of the noise magnitude, the standard deviation was calculated in the same area.

### CT number accuracy

For the determination of the CT numbers, the Catphan CTP401 module [15] was imaged using the default parameters, reconstructed with 10 mm thick slices. For the CT number of water, one of the NPS acquisitions was used. CT numbers were measured as the mean value over a circular ROI with a diameter of 1 cm using ImageJ and compared with the range given in the ACR CT quality control manual [16].

### Low-contrast visibility

Low contrast can be affected by higher resolution and inherently higher noise at the same dose level. Therefore, the low-contrast objects in the Catphan CTP515 module were imaged using the default parameters in all three modes. As the CTDI_vol_ is higher in the SHR and HR modes for the same tube settings, the tube current was lowered to 210 mA to get the same CTDI_vol_ as for the NR mode. Reconstructions were made with 10 mm slice thickness and 1 mm slice increment. One reader (LO) evaluated the middle slice of the CTP515 phantom using diagnostic monitors in a radiological reading room and determined the number of visible contrast objects for each contrast level. To determine the dose necessary to have the same low-contrast visibility as with the MDCT, a series of acquisitions were made at progressively higher tube currents (260 mA, 280 mA, 300 mA, 330 mA, 370 mA and 400 mA).

## Results

### Spatial resolution

**Fig. 1:**
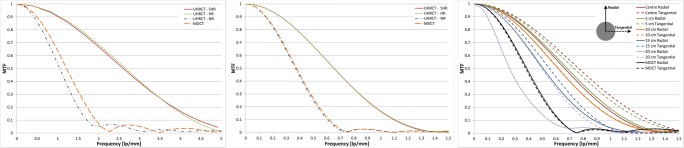
MTFs for UHRCT in SHR, HR and NR modes and MDCT. (Left) Highest possible MTF using step-and-shoot acquisition mode and filtered backprojection. (Middle) MTF in helical mode and reconstructed with FBP (FC08). (Right) MTF of UHRCT under clinical conditions (AIDR3D, FC08) for different distances to the isocentre

Figure 1 and Table 2 show the MTFs of the UHRCT for the three modes and MDCT with filtered backprojection, representing the highest possible resolution. The spatial frequencies obtained with the UHRCT in SHR and HR modes are twice as high as those with the MDCT. The highest resolution of the UHRCT in NR mode is marginally lower than that of the MDCT. Using FBP in helical scan mode, the MTFs of the MDCT and the UHRCT in NR mode are equivalent.

**Table 2:** Frequencies at which the MTF reaches 90%, 50%, 10% and 2% for the UHRCT in SHR, HR and NR modes and for the MDCT

MTF (%)	Frequency (lp/mm)			
	UHRCT			MDCT
	SHR	HR	NR	
90	1.0	1.1	0.4	0.5
50	2.6	2.7	1.0	1.2
10	4.1	3.9	1.7	1.9
2	4.8	5.2	2.9	2.2

**Fig. 2:**
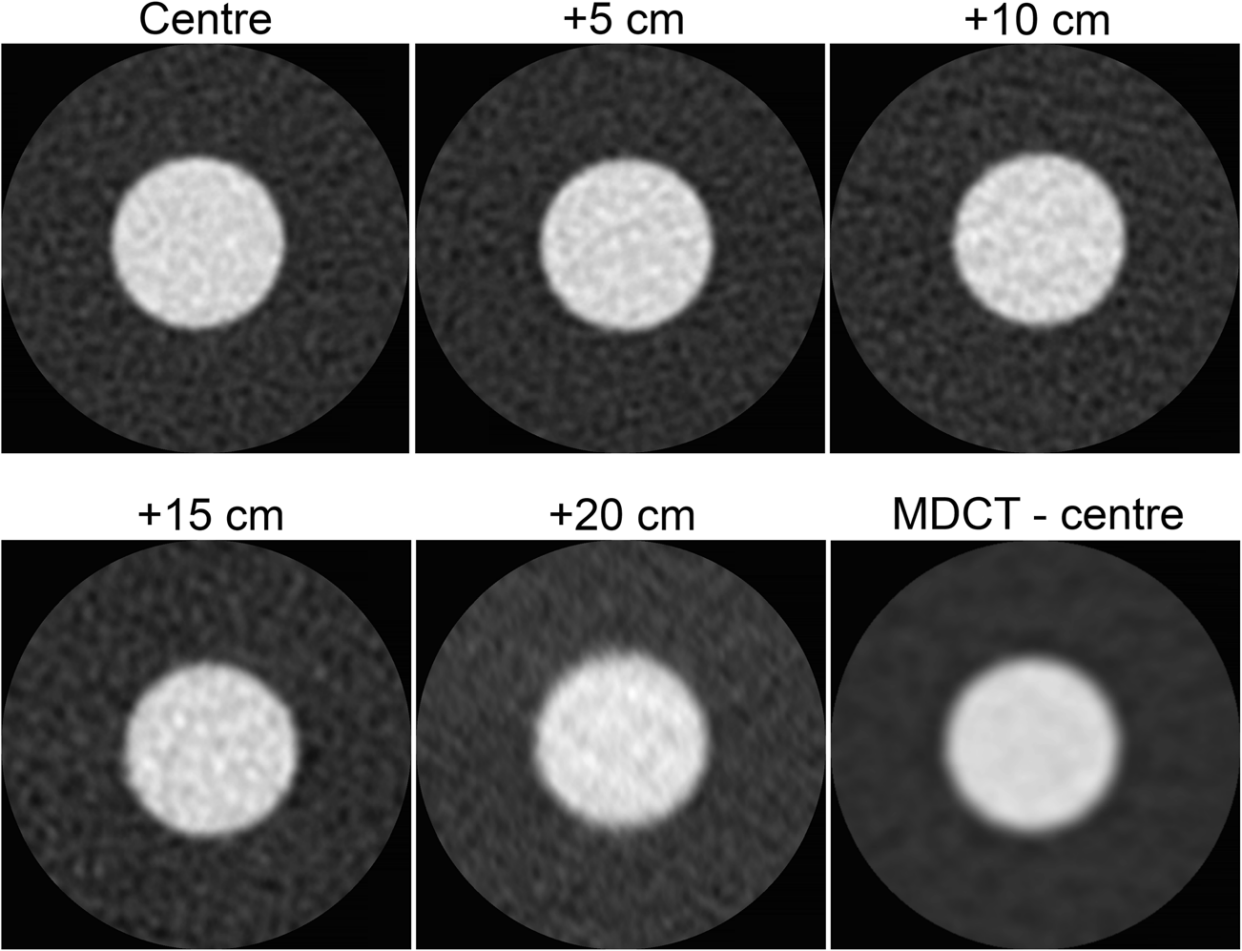
Images of the Teflon rod at different distances from the centre for the UHRCT. The rod imaged by the MDCT at the isocentre of the MDCT system is shown as reference

The resolution change from the isocentre can be seen in Fig. 2. The edges of the Teflon rod are sharper in the isocentre. The MTFs resulting from these edges are plotted in Fig. 1. As can be seen, the UHRCT MTF at the centre is about twice as high as that of the MDCT. Although the UHRCT MTF decreases continuously with increasing distance from the isocentre towards the periphery of the field of view, it remains higher than that of the MDCT, except at the farthest point measured: 20 cm from the isocentre in the radial direction.

### Slice sensitivity profile

In HR mode, the SSP is comparable to the MDCT, having a FWHM of 0.79 mm and 0.77 mm, respectively. For the UHRCT SHR mode, the SSP is narrower. The FWHM of the SHR mode, 0.45 mm, is about 1.7 times smaller than that of the MDCT.

### CT number uniformity

The differences of the CT number value of each peripheral ROI were within the 5 HU limit from the centre ROI, as specified in the ACR protocol [16].

### Noise

**Fig. 3:**
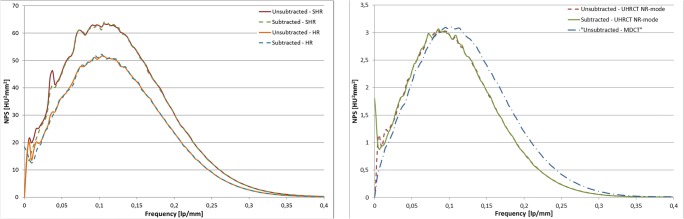
Subtracted and unsubtracted NPS for the UHRCT in (left) HR and SHR modes and (right) for the NR mode and MDCT. Images are reconstructed using AIDR 3D enhanced reconstruction technique with FC08 filter kernel

The noise magnitudes for the SHR, HR, NR and MDCT were (average ± one standard deviation) 30.0 HU ± 0.3 HU, 27.0 HU ± 0.3 HU, 21.9 HU ± 0.3 HU and 24.3 HU ± 0.3 HU, respectively. The NPS for the different modes are shown in Fig. 3. The peak frequency of the HR and SHR was 0.11 lp/mm. For the NR mode and the MDCT, the peak frequencies were 0.08 lp/mm and 0.09 lp/mm, respectively.

### CT number deviation

CT numbers were found to be all within the expected range as defined in the ACR protocol [16] except for the LDPE in NR mode (Table 3), which was somewhat lower than expected and lower compared with the SHR and HR modes (− 81 versus − 87).

**Table 3:** The CT numbers resulted from different materials and scan modes. Used phantoms and expected range by the ACR [17] is given in the last column

CT number					
Material	Phantom	SHR (HU)	HR (HU)	NR (HU)	Exp. range (HU)
Air	Catphan 500	− 981	− 982	− 976	− 1005 to − 970
LDPE	Catphan 500	− 87	− 87	− 81	− 107 to − 84
Water	320 mm water phantom	− 1.0	− 0.9	− 0.4	− 7 to 7
Acrylic	Catphan 500	125	124	127	110 to 135
Teflon	Catphan 500	898	897	920	850 to 970

### Low-contrast visibility

**Fig. 4:**
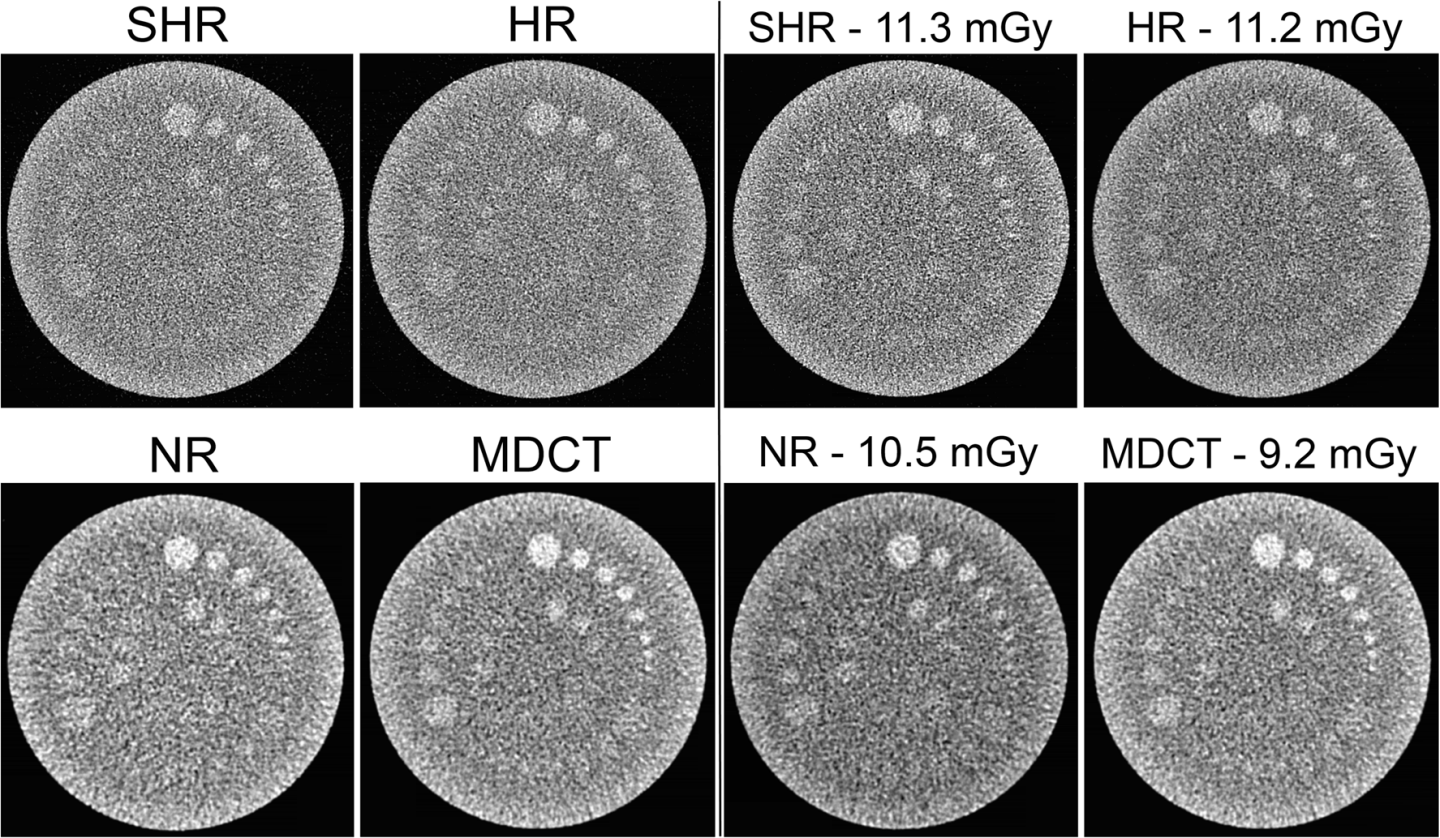
Images of the Catphan low-contrast objects in the CTP515 module acquired in NR, HR and SHR modes of the UHRCT and on the MDCT (left) at a CTDI_vol_ of 9.1 mGy and (right) for the same low-contrast detectability, the CTDI_vol_ is noted. Note that the window width is not the same for all images; for the SHR and HR modes, it is 60 (left) and 50 (right) and for the NR and MDCT it is 30

The low-contrast visibility is best for the MDCT. The 0.5% contrast objects were less visible in the UHRCT NR mode images than in the MDCT ones. For the UHRCT SHR and HR modes compared with MDCT, the edges of the low-contrast object were more sharply delineated (Fig. 4). In Tables 4 and 5, the number of visible contrast objects is given. The extra dose necessary for the UHRCT NR mode to have same low-contrast object visibility was 14%. For the HR and SHR modes, this dose increase was approximately 23%.

**Table 4:** Number of visible supra slice contrast objects in the Catphan CTP515 module

Contrast (%)	SHR (HU)	HR (HU)	NR (HU)	MDCT (HU)
1	7	8	7	8
0.5	6	5	5	7
0.3	2	2	3	3

**Table 5:** Number of visible sub slice contrast objects in the Catphan CTP515 module

Z-axis length (mm)	SHR (HU)	HR (HU)	NR (HU)	MDCT (HU)
7	2	2	2	3
5	1	2	2	2
3	0	0	1	1

## Discussion

**Fig. 5:**
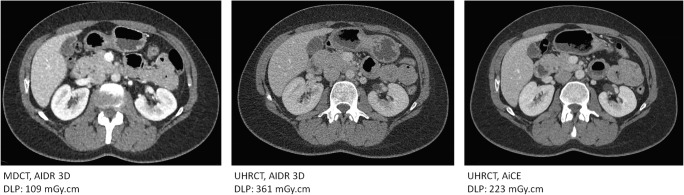
Three axial venous phase contrast enhanced abdominal follow-up CT scans of a female patient with a resected stomach tumour at different time-points. (Left) MDCT at 100 kV with hybrid-iterative reconstruction (middle) UHRCT with HR mode at 120 kV with hybrid-iterative reconstruction and (right) UHRCT with HR mode at 120 kV with deep learning image reconstruction. Despite different contrast timing and tube potential compared with MDCT, the UHRCT using AIDR has better delineation of (retroperitoneal) structures, but more (fine grained) noise. Using deep learning image reconstruction with UHRCT results in even better delineation of the retroperitoneal structures and mesenteric vessels, with less perceived noise than in the other scans

In this study evaluating the physical properties of UHRCT, we found that spatial resolution in SHR and HR modes is about twice as high as that of the MDCT at the isocentre. Spatial resolution away from the isocentre remains higher than the central resolution in MDCT, except at the outermost periphery. In Fig. 5, an example of separate abdominal scans of the same patient on MDCT and on UHRCT, using the HR mode, is shown. In this figure, it can be seen that the UHRCT acquisition results in a better delineation of structures.

The finding that resolution falls off towards the periphery in UHRCT is a known phenomenon and generally observed in CT. This drop in resolution as a function of distance from the centre is likely caused by the finite number of views and focal spot size. In UHRCT, the relative drop in resolution is comparable to that of other systems [17–19].

As a result of the non-linear behaviour of the hybrid-IR reconstruction technique, the noise magnitudes for the high-resolution modes are lower than would be expected given the noise magnitude in NR mode and of the MDCT. Appendix B shows the noise magnitude and texture as a function of dose for hybrid-IR and FBP reconstruction techniques for the different resolution modes. It can be seen that for the former technique noise magnitude is rather constant over a large range of dose values. However, noise structure becomes grainier with decreasing dose, while with FBP, noise structure remains the same. Using hybrid-IR, resolution is maintained up to a certain point, beyond which resolution is affected too. As expected, this effect is not observed using FBP reconstruction.

The NR mode of UHRCT is comparable with the MDCT with respect to CT number, resolution and uniformity. Using an abdominal soft tissue kernel, the noise in NR mode has a lower magnitude than with the MDCT and lower high-frequency components. This might be caused by the iterative reconstruction algorithm using different internal optimisations and suppressing more high-frequency noise in NR mode on the UHRCT compared with on the MDCT, although the user settings are the same for both reconstructions.

In SHR and HR modes, the CTDI_vol_ is higher than that for the NR mode for the same tube settings. This is caused by this UHRCT changing the wedge filter automatically when one of the high-resolution modes is used, resulting in somewhat softer spectra in the high-resolution modes.

Low-contrast detectability is a little lower in the NR mode compared with MDCT, but only a slight dose increase results in the same low-contrast detectability. At our institution, radiation exposure of abdominal MDCT scans is below the achievable levels used in many countries [20, 21]; the median (1st and 3rd quartiles) of the CTDI_vol_ and DLP used clinically over the last year on our MDCT was 3 mGy (2.3–4.8 mGy) and 155 mGy cm (116–252 mGy cm), respectively. The slight dose increase necessary to achieve the same low-contrast detectability with the UHRCT in high-resolution modes will not raise the dose above achievable levels of the diagnostic reference levels of 11 mGy and 550 mGy cm.

The low-contrast detectability of the high-resolution modes is lower than that of the MDCT at the same CTDI_vol_; to make it comparable, an extra dose of about 23% is needed. However, in clinical practice, the dose used in HR mode is about three times higher than that for NR mode (see also Fig. 5). This is due to the fact that the automatic exposure control aims to keep the noise magnitude constant assuming FBP reconstruction. Since in HR mode the in-plane resolution is twice as high compared with that in the MDCT, it would be expected that a fourfold increase in dose would be needed. The lower observed factor is, in addition to other optimisations, mainly due to a more efficient detector layout, with reduced septa thickness. Lowering the dose to a level that would result in the same low-contrast detectability would lead to a level of high-frequency noise that would probably be unacceptable to radiologists. However, a clinical performance–based assessment of the dose increase required with HR mode has not yet been performed. New deep learning reconstruction techniques (AiCE reconstruction, Canon Medical Systems Corporation) [22] aim to lower the noise while maintaining resolution. In our experience, this technique has a higher impact on higher noise conditions, such as in HR mode at low dose, than in NR mode. Therefore, it allows to achieve scans in HR mode at half the achievable level of the DRL (see Fig. 5).

**Fig. 6:**
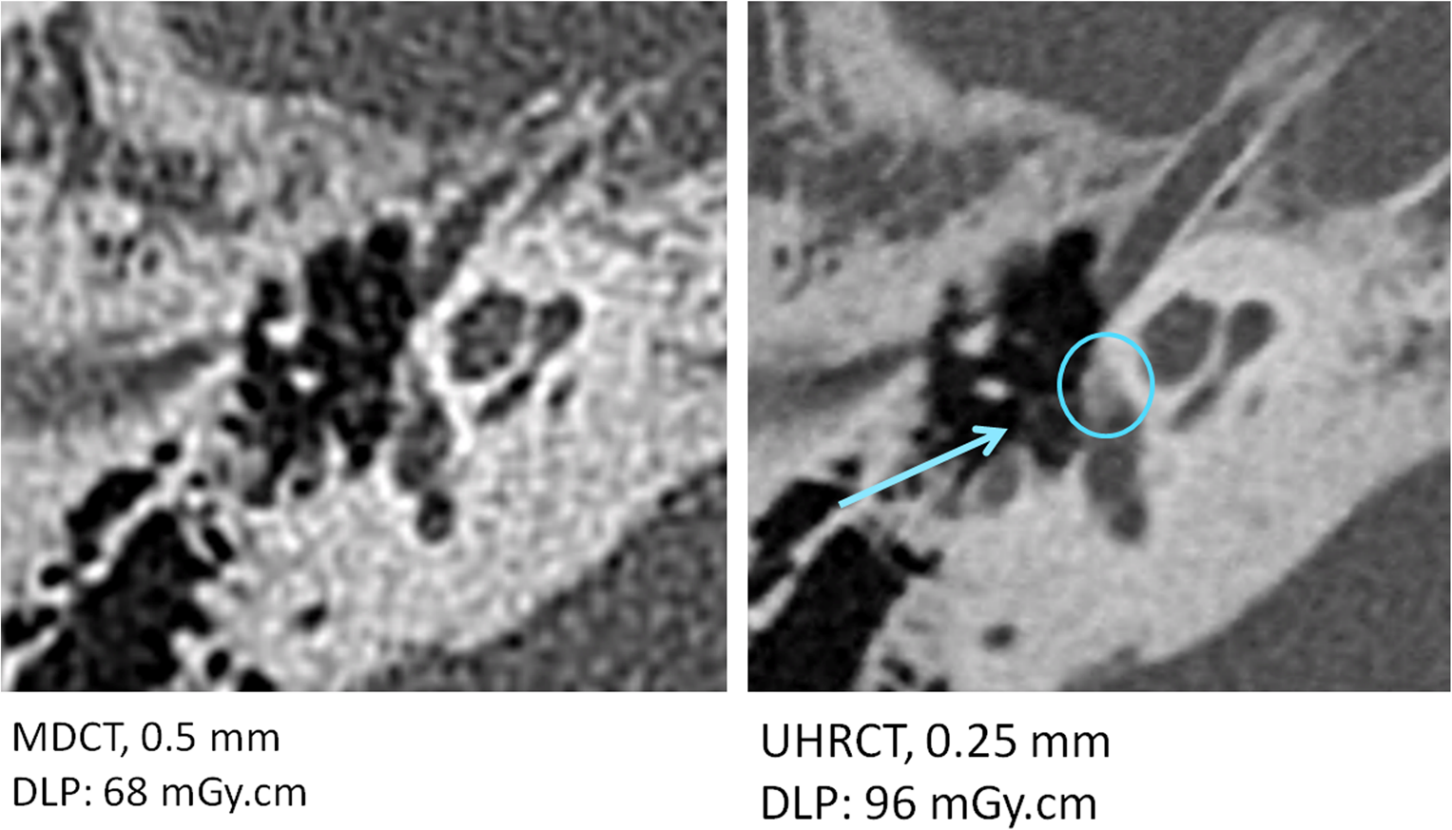
Two different right ear bone kernel CT scans of fenestral otosclerosis as a subtle, focal area with lucency in front of the stapedial footplate (circle). (Left) MDCT and (right) UHRCT in HR mode, both using hybrid-iterative reconstruction. The UHRCT scan contains less noise and enables better delineation of the stapes (arrow), the trabeculae of the medullary bone of the mastoid, the bony otic capsule and the focal lucency (circle) in this capsule representing the disease

Our study has several limitations. First, most measurements were performed with an abdominal soft tissue kernel only. Although it is not a high-resolution kernel, it is the most commonly used kernel at our institution. As the system resolution is higher than the resolution at abdominal settings, it is expected that high-resolution kernels could also benefit from the higher resolution capabilities. For example, Fig. 6 shows an inner ear scan reconstructed with a high-resolution kernel. The additional detail in the UHRCT reconstruction, enabling better delineation of the structures, can be appreciated.

Second, the low-contrast measurements were only performed in a qualitative fashion without performing an extensive observer study. Low-contrast details were evaluated while the observer knew where they should be. However, this method is expected to result in a good first-order estimation of the low-contrast capabilities, since any bias will probably affect the results in the same manner for all conditions. More elaborate studies would need to be performed to study low-contrast visibility in more detail.

Finally, some caution should be used when interpreting linear metrics, such as the MTF and NPS, when evaluating images reconstructed with non-linear algorithms, such as the AIDR3D algorithm used here. The resulting spatial resolution and noise characteristics in different areas containing signals of different characteristics, even across the same image, could vary, and not be well represented by a single MTF or NPS. However, having performed this comparison across acquisition modes and between the two systems using the same testing tools and phantoms does ensure that the relative differences, and similarities, in performance found are reflective of the actual system/algorithm capabilities.

In conclusion, the HR and SHR modes of the UHRCT system result in double the in-plane spatial resolution of the MDCT system, while the NR mode is comparable to that of the MDCT. The trade-off is that about 23% more dose is needed for the same low-contrast detectability. Upcoming deep learning reconstruction techniques are promising in lowering the current clinical dose penalty while maintaining the spatial resolution.

## Electronic supplementary material

ESM 1 (DOCX 189 kb)

## Abbreviations

ACR: American College of Radiology
AI: Artificial intelligence
CTDI_vol_: Volume CT dose index
Del: Detector element
ESF: Edge spread function
FWHM: Full width at half maximum
HR: High resolution
LSF: Line spread function
MTF: Modulation transfer function
NPS: Noise power spectrum
NR: Normal resolution
SHR: Super-high resolution
SSP: Slice sensitivity profile
UHRCT: Ultra-high-resolution CT
