# Correction to: Physical evaluation of an ultra-high-resolution CT scanner

**DOI:** 10.1007/s00330-020-06793-x

**Published:** 2020-03-25

**Authors:** Luuk J. Oostveen, Kirsten L. Boedeker, Monique Brink, Mathias Prokop, Frank de Lange, Ioannis Sechopoulos

**Affiliations:** 1 Department of Radiology and Nuclear Medicine, Radboud University Medical Center, P.O. Box 9101, (Route 766), 6500 HB Nijmegen, The Netherlands; 2 Canon Medical Systems Corporation, Otawara, Japan

**Correction to: European Radiology**

10.1007/s00330-019-06635-5

The original version of this article, published on 10 February 2019, unfortunately contained a mistake. The axes of the graphs in Fig. 3 are incorrect. The correct figure is given below. Therefore, the last two sentences in “Results”, section “Noise”, should read: “The peak frequency of the HR and SHR was *0.21 lp/mm*. For the NR mode and the MDCT, the peak frequencies were *0.17 lp/mm* and *0.21 lp/mm*, respectively.”

**Fig. 3:**
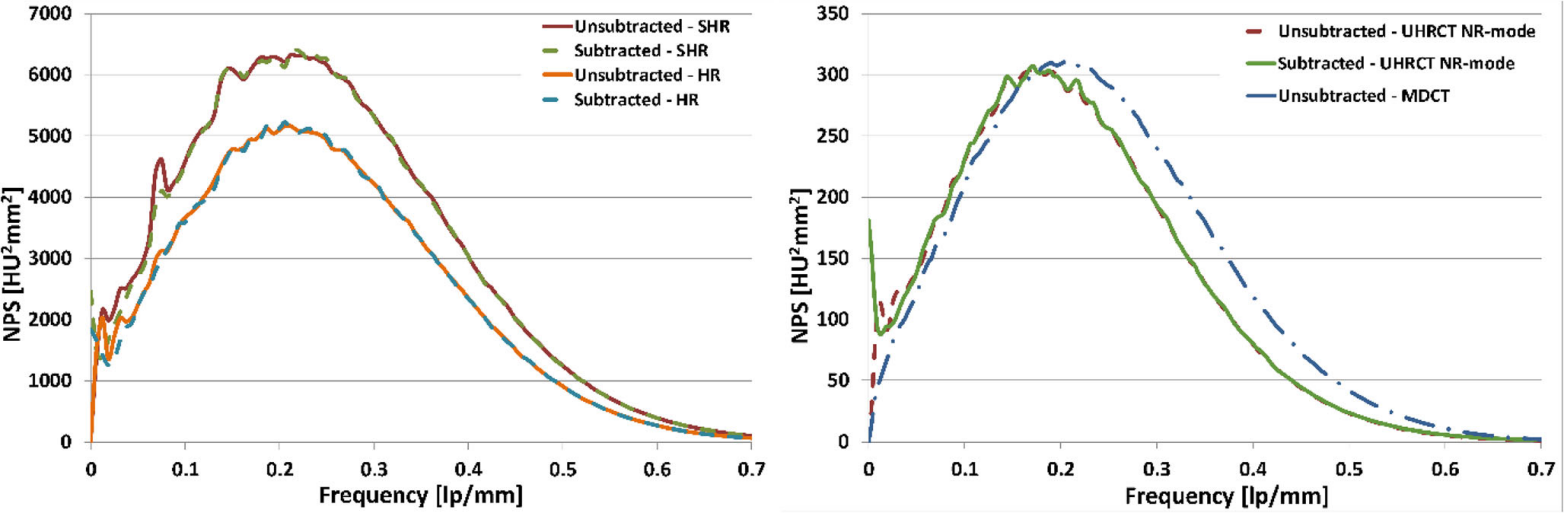
Subtracted and unsubtracted NPS for the UHRCT in (left) HR and SHR modes and (right) for the NR mode and MDCT. Images are reconstructed using AIDR 3D enhanced reconstruction technique with FC08 filter kernel

**Fig. B2:**
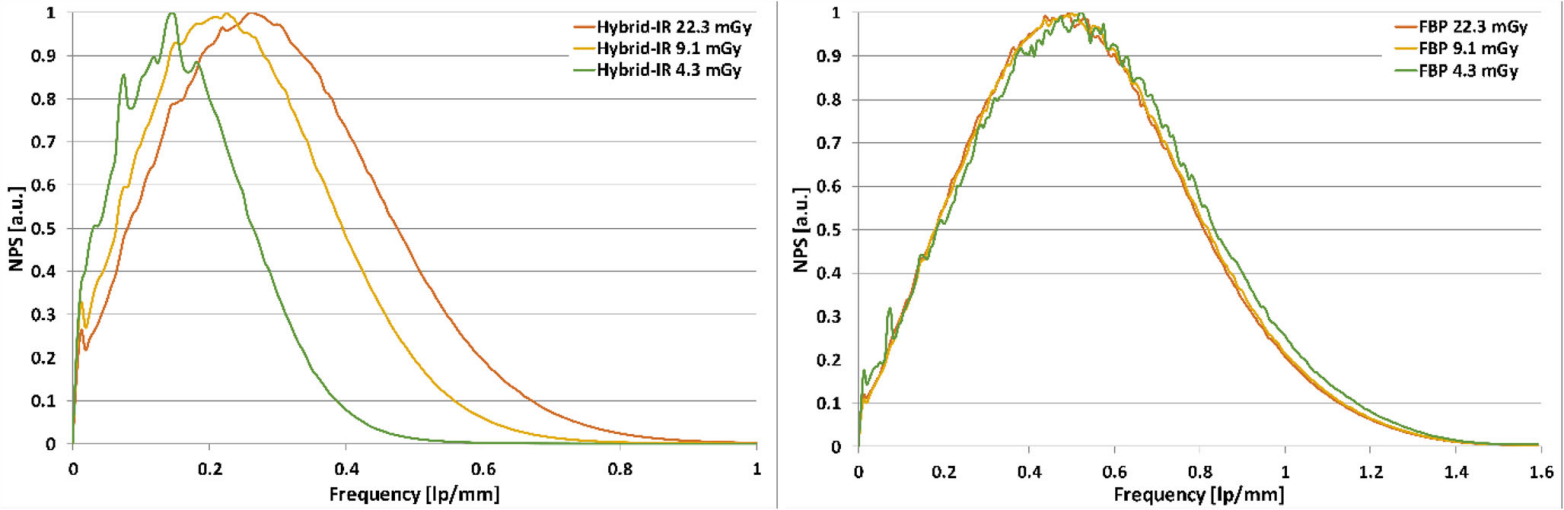
Normalised NPS for (left) Hybrid-IR (AIDR 3De) and (right) FBP for different dose settings in HR resolution mode

The X-axis of the graphs in Fig. B2 (Addendum B) has the same issue. The correct figure is given below. These changes do not affect any conclusion drawn from the results.

