# Supplementary material for: Physical evaluation of an ultra-high-resolution CT scanner
Source: Eur Radiol. 2020 Feb 10;30(5):2552–60. doi: 10.1007/s00330-019-06635-5 (PMC7160079; doi:10.1007/s00330-019-06635-5)
Supplement: Supplementary file 1 — (DOCX 189 kb) [file 330_2019_6635_MOESM1_ESM.docx]

**Supplementary Materials**

**Appendix A**

In this appendix the methods for determining the modulation transfer function (MTF) and noise power spectrum (NPS) are explained in further detail.

Determining MTF with filtered backprojection reconstruction technique

To determine the MTF a 50 μm diameter tungsten wire fixed in a frame of balsa wood was imaged. A 20 mm FOV around the wire was reconstructed using filtered backprojection.

A script (MATLAB R2014b, The MathWorks, Inc.) was created to calculate the MTF as follows: the background HU value was determined using voxels at a distance between 150 and 200 pixels from the maximum value. This value was subtracted from all voxel values. A square of 200 by 200 voxels in the centre was Radon transformed to get the line spread function (LSF). The LSF was cut at the first zero crossing and zero-padded to 128 pixels. The magnitude of the Fourier transform of the LSF was taken to get the MTF. Finally, the MTF was Bessel corrected for the finite size of the wire[1].

Determining MTF with Hybrid iterative reconstruction technique

Due to the Hybrid iterative reconstruction a thin wire can’t be used as this reconstruction technique would diminish such a small delta pulse. Therefore the edge of a cylindric object is used to calculate the MTF using a procedure comparable to that described by Richard et al[2], as follows. The centre of mass of the cylindric object was found and the edge spread function (ESF) through the centre of mass was determined in the tangential and radial directions. These steps were repeated for every slice containing the cylindric object. The average ESF was calculated by averaging over all ESFs using the subpixel position of the half maximum as the reference point. The LSF was calculated by numerical

differentiation of the averaged ESF with a standard central-difference algorithm. Finally, the magnitude of the Fourier transform of the LSF was taken to get the MTF.

Determining NPS

The NPS was calculated following the procedure described by Boedeker et al[3]. For SHR acquisitions a central ROI of 256 by 256 pixels was used, while for the NR and MDCT acquisitions 128 by 128 pixels were used. The average value within the ROI was subtracted from the pixel values to get the unsubtracted NPS. For the subtracted NPS the pixel values in a ROI at the same location of an arbitrary scan were subtracted and the result divided by the square root of 2, to correct for the additional noise due to the subtraction. The area was zero-padded to 512 by 512 voxels. The square of the magnitude of the Fourier transform was taken to obtain the two-dimensional NPS. Finally, a one-dimensional NPS was created by radial averaging of the two-dimensional NPS. The frequency at which the NPS peaks was used as a single-value metric for the noise texture[4].

References

1. Nickoloff EL (1988) Measurement of the PSF for a diameter and pixel size. Phys Med Biol 33:149–155

2. Richard S, Husarik DB, Yadava G, et al (2012) Towards task-based assessment of CT performance: System and object MTF across different reconstruction algorithms. Med Phys 39:4115–4122. https://doi.org/10.1118/1.4725171

3. Boedeker KL, Cooper VN, McNitt-Gray MF (2007) Application of the noise power spectrum in modern diagnostic MDCT: Part I. Measurement of noise power spectra and noise equivalent quanta. Phys Med Biol 52:4027–4046. https://doi.org/10.1088/0031-9155/52/14/002

4. Solomon J, Wilson J, Samei E (2015) Characteristic image quality of a third generation dual-source MDCT scanner: Noise, resolution, and detectability. Med Phys 42:4941–4953. <https://doi.org/10.1118/1.4923172>

**Appendix B**

This appendix is meant to clarify the relationship between noise magnitude, measured as standard deviation (SD), and dose for the different reconstruction algorithms. For filtered backprojection (FBP) it is well known that the reciprocal variance (SD-2) increases linearly with increasing dose. For hybrid iterative reconstruction (Hybrid-IR) this is not necessarily the case; changes in dose can have impact on noise magnitude, noise texture, and resolution.

Methods and materials

The methods and materials used for determining the NPS are repeated with different dose settings, resolution modes, and reconstruction techniques. The settings used are the same as in table 1 of the main manuscript, except that the tube current was changed. In table B1 the different tube current settings, as well as the resulting CTDIvol, are given.


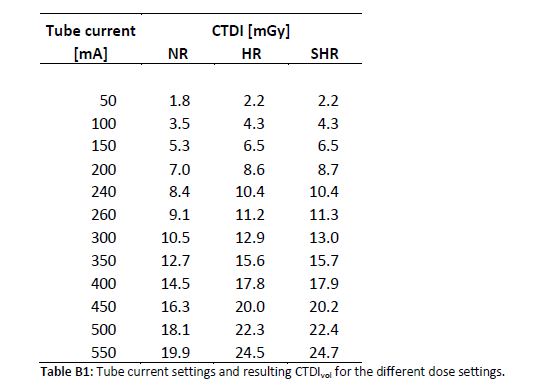


The noise magnitude was determined at each setting listed above, while the NPS and MTF were obtained at tube currents of 100 mA and 500 mA in HR resolution mode for Hybrid-IR (AIDR 3D Enhanced) and FBP reconstructions. For this, the same procedures described in the main manuscript and Appendix A: Determining MTF with Hybrid iterative reconstruction technique were used.

Results

Figure B1 shows the noise as a function of dose for the different resolution modes and reconstruction techniques. As can be seen, FBP does show the expected linear relationship between the reciprocal variance (SD-2) and dose. The Hybrid-IR doesn’t follow this relationship, except for in NR resolution mode, at a CTDIvol value of 14.5 mGy and above.


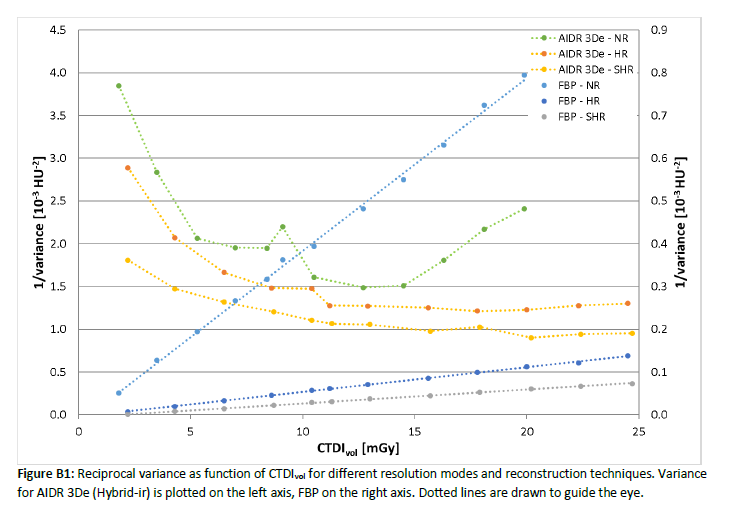


Figure B2 shows the NPS for the HR mode for the two settings calculated for this appendix, and the NPS from the main manuscript (at a CTDIvol of 9.1 mGy). The shape of the NPS remains the same for the FBP technique, but changes for the Hybrid-IR technique. The noise texture becomes grainier with decreasing dose.


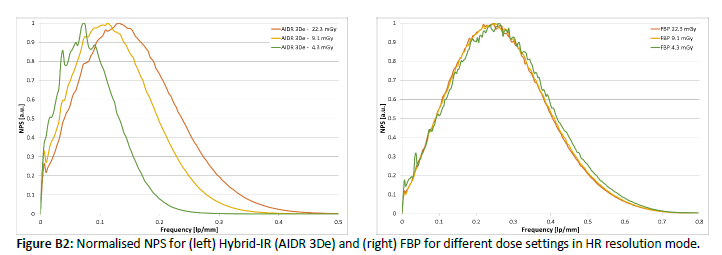


Figure B3 shows the MTF for the different settings. For Hybrid-IR the MTF decreases under low dose conditions, but for FBP the MTF is not influenced by dose.


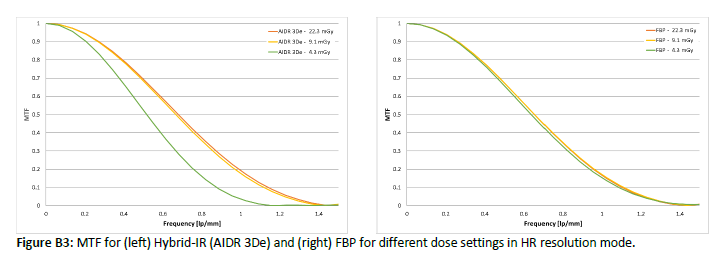


Conclusion

Reconstructions using filtered backprojection result in a linear relationship between reciprocal variance and dose, while the MTF and the shape of the NPS are not affected by dose. For Hybrid iterative reconstruction (AIDR 3D enhanced) this is not the case. If dose is decreased, noise becomes grainier but noise magnitude doesn’t necessarily increase. In low dose situations, the resolution is eventually affected.
